# Sarcopenia: no consensus, no diagnostic criteria, and no approved indication—How did we get here?

**DOI:** 10.1007/s11357-023-01016-9

**Published:** 2023-11-24

**Authors:** William J. Evans, Jack Guralnik, Peggy Cawthon, James Appleby, Francesco Landi, Lindsay Clarke, Bruno Vellas, Luigi Ferrucci, Ronenn Roubenoff

**Affiliations:** 1 University of California, Berkeley, Berkeley, CA USA; 2 University of Maryland School of Medicine, Baltimore, MD USA; 3 California Pacific Medical Center, San Francisco, CA USA; 4 Gerontological Society of America, Washington, DC USA; 5 Catholic University of the Sacred Heart, Milan, Italy; 6 Alliance for Aging Research, Washington, DC USA; 7 Toulouse University Hospital, Toulouse, France; 8 National Institute on Aging, Bethesda, MD USA; 9 Tufts University School of Medicine, Boston, MA USA

**Keywords:** Sarcopenia, Muscle mass, Body composition, Regulatory approval, Functional capacity

## Abstract

In addition to the role of skeletal muscle in movement and locomotion, muscle plays a critical role in a broad array of metabolic processes that can contribute to improved health or risk of disease. The age-associated loss of muscle has been termed sarcopenia. The muscle is the primary site of insulin-stimulated glucose disposal and the largest component of basal metabolic rate, directly and indirectly affects bone density, produces myokines with pleiotropic effect on muscle and other tissues including the brain, and stores essential amino acids essential for the maintenance of protein synthesis during periods of reduced food intake and stress. As such, not surprisingly deterioration of skeletal muscle health, typically operationalized as decline of muscle mass and muscle strength is both a powerful risk factor and main consequence of chronic diseases, disability, and loss of independence, and it is one of the strongest risk factors for mortality. However, skeletal muscle remains one of the most plastic of all tissues, with rapid changes in rates of protein synthesis and degradation in response to physical activity and inactivity, inflammation, and nutritional and hormonal status. This has made the development of pharmacological therapies to increase muscle mass (or prevent loss), an important goal for decades. However, while remarkable advances in the understanding of molecular and cellular regulation of muscle protein metabolism have occurred recently, there are no approved drugs for the treatment of sarcopenia, the loss of skeletal muscle affecting millions of older people. The goal of this paper is to describe the possible reasons for the lack of new and effective pharmacotherapies to treat one of the most important risk factors for age-associated disease and loss of independence.

## Introduction

**Fig. 1:**
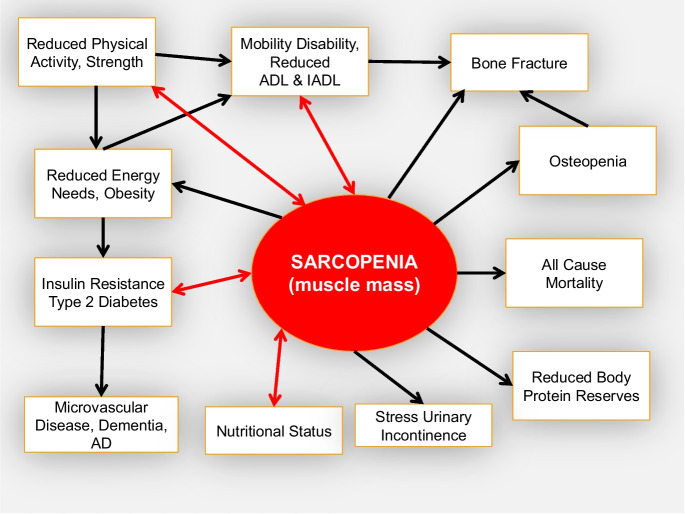
Age-associated loss of muscle mass (sarcopenia [1]) plays a central role in the onset of several age-associated syndromes and diseases which, in turn, affects the risk of a cascade of disorders typically associated with advancing age [3, 44–48]. [49]

Changing body composition is, perhaps, the most prominent and obvious feature of advancing age. Body fatness increases even in men and women who remain weight stable and physically active as they grow older, the density of bones decreases, and the amount of skeletal muscle declines. More specifically, the geriatric syndrome, sarcopenia, was originally defined as the age-associated loss of skeletal muscle mass (Fig. 1) [1] that was hypothesized to increase the risk of functional decline and disability. In the years following that initial definition, an accurate assessment of whole body muscle mass has proven to be elusive, and most investigators have used dual X-ray absorptiometry (DXA) or bioelectric impedance (BIA) assessments of lean body mass (LBM) as a surrogate measurement in large cohort studies. However, muscle mass is only one component of LBM that also includes body water, viscera, fibrotic, and connective tissue.

To date, cross-sectional and longitudinal aging cohort studies have reported little or no relationship between low LBM and increased risk of health-related outcomes, including functional capacity, disability, and mortality. A meta-analysis [2] of longitudinal observational studies in older people (≥ 65 years) conducted between 1976 and 2012 examined reported data of body composition (BIA, DXA, CT) and physical functional capacity. In the studies that examined lean mass (which is incorrectly termed “muscle mass”), the authors concluded that “low muscle mass was not significantly associated with functional decline.” They also concluded that the role of muscle mass in the development of functional decline was unclear but was “much smaller than the role of fat mass and muscle strength.” While there is no disagreement among experts that skeletal muscle mass is diminished with advancing age, the degree to which this reduction is associated with loss of functional capacity and risk of disability has only recently been described [3]. The D_3_Creatine dilution method has recently emerged as an accurate measurement of muscle mass. As described by Orwoll et al. [4], the muscle measured by this method represents only about 50% of LBM in a population of older men and is strongly associated with functional status. The incorrect assumption that LBM is an accurate measurement of muscle mass has resulted in incorrect conclusions on the importance of muscle mass in defining sarcopenia. However, at the present time, there are no recognized diagnostic criteria for sarcopenia, despite an assigned ICD-10-CM code (M62.84). Although sarcopenia is recognized as a common geriatric syndrome, the lack of a consensus on its definition is one factor that has resulted in inaction by regulators in recognizing sarcopenia as a treatable geriatric syndrome, which has, in turn, diminished enthusiasm for drug development. Importantly, approval as a treatable indication is not only necessary for prescription and reimbursement by third-party payers for drugs but also for reimbursement for rehabilitation involving prescribed exercise programs in the USA. The positive effects of exercise in frail, weak sarcopenic men and women are well-described; however, reimbursement by third party payers including Medicare to treat sarcopenia is not available.

## Muscle mass and age

Decrease in skeletal muscle mass with advancing age has a complex etiology, occurs slowly, and affects all humans, even those who are highly physically active and well-nourished [5]. Larsson et al. [6] have provided a comprehensive review of molecular, neurological, cellular, and metabolic factors associated with sarcopenia. There is a well-described decrease in size and amount of type II fibers with advancing age that may account for decreases in maximal sprinting capacity or rapid, explosive power in athletes and decreased maximal force production in all men and women with advancing age. Larsson et al. [7] described a selective atrophy of type II fibers with a relative preservation of type I fibers with advancing age. Multiple pathways lead to reduced rates of muscle protein synthesis and mass with age, including growing insulin resistance (secondary to increased body fat and inactivity), inflammation, reduced testosterone, and growth hormone production.

While the age associated loss of skeletal muscle takes place slowly over much of the adult lifetime [8], the loss is greatly accelerated in several circumstances, particularly in older men and women. Two of the most prominent causes are bed rest (extreme inactivity) and cachexia (loss of skeletal muscle secondary to chronic disease) [9]. Ten days of bed rest in healthy older people (67 ± 5 years) resulted in a 30% reduction in the rate of muscle protein synthesis, with loss of almost one kg of leg lean mass and 15·6% of leg strength [10]. This loss of leg lean mass is almost threefold greater than that seen in healthy young men and women after 28 days of bed rest [11, 12]. These dramatic losses of muscle mass in a such short periods of time, typical of an in-patient hospitalization, often result in a catastrophic loss of strength, function, and independence. The loss of lean mass, muscle mass, and strength during hospitalization in older patients can be restored through targeted rehabilitation [12]; however, due to a lack of a specific indication, the cost of rehabilitation is often not covered by third party payers. New potential therapies and potential drug targets are in development to treat weakness and health in older people [13], however none specifically to treat sarcopenia for reasons outlined here.

## Defining sarcopenia

Several definitions and consensus panels have attempted to describe diagnostic criteria for sarcopenia [14–25]. Sarcopenia has enormous societal consequences and has been assigned an ICD-10-CM code, M62·84, but with no specific diagnostic criteria. Mayhew et al. [26] recently determined the degree to which published consensus definitions of sarcopenia were in agreement using cross-sectional data from 10,820 men and women (age 65–85 years) participating in the Canadian Longitudinal Study on Aging who had data required to diagnose sarcopenia. In this study, sarcopenia was defined as (1) low lean mass alone, (2) low lean mass and low muscle strength, (3) low lean mass and low physical function, and (4) low muscle strength and low physical function. Grip strength was chosen as the measure of muscle strength and gait speed as the measure of physical function because they are recommended by each of the consensus definitions. They reported that the combination of variables used to determine sarcopenia and many of the lean body mass adjustment techniques have insufficient agreement to be considered equivalent and concluded that a unified definition of sarcopenia is required. The lack of agreement of criteria for the diagnosis of sarcopenia remains a major obstacle for regulators and healthcare professionals to identify and treat sarcopenia in older men and women. The Global Leadership in Sarcopenia Steering Committee published a consensus document to standardize the definitions of terms that have previously been proposed to be related to sarcopenia [27]. However, harmonization of definitions of terms is unlikely to result in an operational and acceptable definition of sarcopenia.

A recent survey [28] of 253 practicing US physicians (Internal Medicine, Family Medicine, Geriatrics, and Physical Medicine & Rehabilitation (PM&R)) reported that less than 20% of internists and family medicine physicians were very familiar with the term sarcopenia (70% for geriatricians and 41% of PM&R physicians). More than 75% of those surveyed used no criteria for a diagnosis of sarcopenia. Importantly, when the physicians in the survey were asked what they thought were the most common reasons patients failed to address losses in strength and function, 56% indicated the belief that sarcopenia is a natural component of aging, 41% stated the lack of desire or ability of their patients to change habits with diet or exercise, and 38% said lack of understanding that sarcopenia is treatable. As of mid-2023, a search of the published literature revealed that more than 18,000 references used the term sarcopenia and more than 6800 articles used sarcopenia in the title. Although sarcopenia is one of the most studied geriatric syndromes, there remains no real consensus on how it should be diagnosed, which we believe greatly limits the incorporation of sarcopenia into comprehensive clinical care for older persons and reduces development of new pharmacological therapies for this condition. In particular, the Food and Drug Administration has not recognized sarcopenia as a treatable indication. There are several potential reasons for the lack of consensus on specific diagnostic criteria for sarcopenia and FDA inaction.

1. Lack of a method to measure muscle mass in large cohort studies. The initial definition of sarcopenia was the age-associated decrease in skeletal muscle mass [1]. However, investigators have used LBM or appendicular lean mass (ALM) as surrogate measurements of muscle mass, and because age-associated changes in these measures are not associated with age-associated loss of functional capacity, the conclusions of these studies were that losses of muscle mass are not causally linked to the risk of late-life disability or other health-related outcomes. Longitudinal data from the Health, Aging and Body Composition (Health ABC) study showed a greater rate of decrease in muscle strength than LBM [29], while a more recent study, which actually measured muscle mass, showed that in older men longitudinal changes in muscle mass and strength are significantly related and similar in magnitude [30]. Investigators using LBM and/or ALM often inaccurately refer to these measurements as muscle mass and the general conclusions that follow-on from these observations has been that some, as yet unexplained feature of muscle quality must be responsible for age-related health related outcomes.
2. Lack of consensus or agreement for a definition of sarcopenia. The lack of data on the effects of age-associated loss of muscle mass on health-related outcomes has resulted in the use of muscle strength and/or functional measurements to define sarcopenia. A consortium of experts [21] concluded “Appendicular lean mass… either absolute or scaling for body size, is not a good predictor of adverse health-related outcomes such as mobility limitation, falls, ADL disability, and mortality in community-dwelling older adults.” And “lean mass measured by DXA should not be included in the definition of sarcopenia.” Interestingly, 40% of the consortium disagreed with these statements. In contrast, the data showing that strength and measures of physical functional capacity are significantly associated with health-related outcomes including the risk of disability are far more compelling. As a result of the existing data, Manini [31] described “dynapenia” as the age-associated loss of strength as far more important for risk of disability than loss of muscle mass (only assessed using LBM). A meta-analysis of observational studies [32] in older men and women found that the prevalence of sarcopenia varied between the various definitions ranging from 5 to 17%. They also found that according to the tool being used to assess muscle mass (only LBM), strength, and physical performance, prevalence rates of sarcopenia also varied within definitions (0–22%). The authors concluded that “The establishment of a unique definition for sarcopenia, the use of methods that guarantee an accurate evaluation of muscle mass and the standardization of measurement tools are necessary to allow a proper diagnosis and comparison of sarcopenia prevalence.” As a result, multiple definitions of sarcopenia exist, and there remains no real consensus on what it is or how it should be diagnosed.
3. No systematic measurement of the functional status of older patients. Functional capacity and strength are strongly associated with health-related outcomes in older men and women. A key step for identification of early changes in function should be the establishment of routine and reimbursable assessments of physical function in geriatric patients. At the present time, in the USA, there is no Centers for Medicare and Medicaid Services (CMS) approved reimbursement for a healthcare provider to perform even the most basic of standardized functional tests or quality of life assessments to determine the functional status of their patients. This is critical to identify early changes in strength, gait speed, balance, or endurance of a patient that may be treated with exercise, diet, or new drugs to treat sarcopenia. In particular, the Short Physical Performance Battery (SPPB) is simple and can be performed with a minimum of space and training (www.SPPBguide.com). CMS reimbursement for this test will help to identify large numbers of older patients who may be exhibiting small losses of physical function that may otherwise go unnoticed. In this way, the causes may be identified and treated immediately before any further loss is experienced. The SPPB is a combined functional measurement of usual walking speed, time to stand up and sit down from a chair five times, and standing balance and is associated with risk of disability, institutionalization, and death [33, 34]. Usual walking speed, one of the components of the SPPB, is strongly and independently associated with health-related outcomes and mortality risk [35]. This simple assessment is so strongly linked to health-related outcomes in geriatric patients that it has been termed the “sixth vital sign” [36]. The SPPB (or usual walking speed) can be easily measured in a limited amount of space with no requirement for specialized equipment. In addition, the Functional Assessment of Chronic Illness Therapy (FACIT) is a brief, standardized set of 13 questions [37] that can potentially identify functional changes in patients. Although these measurements are strongly linked to outcomes, they are not commonly assessed in patients older than 65 years. Perhaps because these functional tests are not a routine component of a geriatric assessment by most healthcare providers, early decreases in function are not often recognized and rarely, if ever, used to diagnose sarcopenia. We strongly recommend the routine use of a standardized assessment of functional capacity and fatigue during routine office visits so that such deficits may be quickly identified, and appropriate therapies may be implemented. We also recommend a reimbursement strategy for CMS payment for healthcare practitioners performing the SPPB or habitual gait speed on any patients they deem appropriate.
4. Lack of patient advocacy. Although sarcopenia directly affects the risk of disability, loss of independence, and mortality of millions of older people, there is little public awareness of the condition or pressure to find safe and effective pharmacological therapies. Perhaps, most important may be that many, including some healthcare professionals, believe that sarcopenia and loss of functional independence are natural and normal components of aging. However, previous research in frail, older subjects have demonstrated that components of sarcopenia (strength and low functional status) are responsive to a combination of diet, physical activity, and nonpharmacological interventions [38, 39]. Increased awareness of sarcopenia, its consequences, and its treatability could have a strong impact on improved patient care and interventions. An example of this can be seen in advocacy for increasing research funding in Alzheimer’s disease, which has resulted in congressionally mandated funding of more than $3 billion and the establishment of powerful advocacy groups such as the Alzheimer’s Association (alzfdn.org), with a reported annual revenue of more than $400 million [40]. Because Alzheimer’s disease is a recognized indication for drug development and through powerful patient advocacy, drug development is a high priority for large Pharma and smaller Biotech companies. Patient advocacy groups can be effective in working with congress to greatly increase the awareness of age-related loss of physical function and independence. This effort could be particularly meaningful in demonstrating that treating this problem and keeping older Americans more functional and out of institutional care can help save or reduce the enormous cost of medical and long-term care for geriatric patients. Medicare expenditures are predicted to increase by > 111% between 2019 and 2029 [41] and increasing the number of older people who remain independent resulting from therapies to treat or prevent sarcopenia could have a large effect on reducing these costs.
5. FDA and EMA. Despite decades of research on changes in skeletal muscle amount and function with advancing age, as yet, neither the FDA nor the European Medicine Agency has approved a drug with sarcopenia as the indication. In 2009, a group of researchers and representatives from the National Institute on Aging met with representatives from FDA with the stated purpose of “learning how the FDA evaluates proposals for an indication (that may be specific to geriatrics) and on exploring how several geriatric conditions might conform to this process” [42]. Representatives (Mary Parks, Director of Metabolism and Endocrinology Products and Laurie Burke, Director of Study Endpoints and Label Development) from FDA indicted that “We encourage that qualification efforts begin with reference to a specific disease or condition.. A more specific target population... the intended subset of patients with identified disease or condition.”

The following listing shows the multiple ways that indications may be formulated for the FDA:

21 CFR 201·80(c)( 1 )(i)

The drug is indicated in the treatment, prevention, or diagnosis of a recognized disease or condition, and/or

21 CFR 201·80(c)( 1 )(ii)

The drug is indicated in the treatment, prevention, or diagnosis of an important manifestation of a disease or condition; and/or

21 CFR 201·80(c)( 1 )(iii)

The drug is indicated for the relief of symptoms associated with a disease or syndrome; and/or

21 CFR 201·80(c)( 1 )(iv)

The drug, if used for a particular indication only in conjunction with a primary mode of, e.g., diet, surgery, or some other drug, is an adjunct to the mode of therapy.

However, many problems of aging do not fit well into any of these categories—which requires that a disease, condition, or syndrome be “recognized” before an indication can be approved. Sarcopenia poses a substantial burden to older persons and should be identified as a suitable target for drug development and interventions. The FDA has relied on the consensus by professional organizations that, in turn, publish guidelines for identifying and treating a specific indication. One important step forward is the recognition of sarcopenia with a specific ICD-10 code (M62·84) that healthcare professionals may use for reimbursement. However, the sarcopenia ICD-10, like other ICD codes, has no specific diagnostic criteria. The major professional organizations representing individuals who study aging and provide care to the 65+ population (the Gerontological Society of America, American Geriatrics Society, and the European Geriatric Medicine Society) have not issued guidelines for identification and treatment of sarcopenia—a critical step toward FDA recognition of sarcopenia.

## Conclusions

Sarcopenia is a complex geriatric syndrome that is often observed in older men and women with multiple concurrent diseases. As a result, they are often excluded from clinical trials in an effort to reduce variability and ensure specificity of efficacy. However, without specific guidelines on how to diagnose and treat sarcopenia in these patients, sarcopenia will remain unrecognized and poorly understood by most healthcare professionals. As the population ages, patients experiencing the loss of muscle and function that is associated with chronic conditions will continue to increase. Perhaps, more than any other age-associated disorder, sarcopenia directly contributes to loss of independence. More specifically, muscle mass is strongly associated with risk of disability and mortality in older people, and it may be time to return to the initial definition of sarcopenia as low muscle mass and function. Hardee and Lynch [43] wrote “Debate and many (not-so-helpful) publications regarding nuances around specific definitions have restricted progress in accepting, understanding and treating sarcopenia.” We strongly recommend coordination of efforts of public advocacy groups, geriatrics focused professional societies, regulators, researchers, and federal agencies to agree upon and implement primary outcome measurements in sarcopenia trials that FDA and EMA will support. In particular, professional societies must lead the efforts to raise public and medical awareness that sarcopenia is not a natural consequence of aging and may potentially be prevented and treated.
